# Association Testing of Clustered Rare Causal Variants in Case-Control Studies

**DOI:** 10.1371/journal.pone.0094337

**Published:** 2014-04-15

**Authors:** Wan-Yu Lin

**Affiliations:** Institute of Epidemiology and Preventive Medicine, College of Public Health, National Taiwan University, Taipei, Taiwan; University of California, Irvine, United States of America

## Abstract

Biological evidence suggests that multiple causal variants in a gene may cluster physically. Variants within the same protein functional domain or gene regulatory element would locate in close proximity on the DNA sequence. However, spatial information of variants is usually not used in current rare variant association analyses. We here propose a clustering method (abbreviated as “*CLUSTER*”), which is extended from the adaptive combination of *P*-values. Our method combines the association signals of variants that are more likely to be causal. Furthermore, the statistic incorporates the spatial information of variants. With extensive simulations, we show that our method outperforms several commonly-used methods in many scenarios. To demonstrate its use in real data analyses, we also apply this *CLUSTER* test to the Dallas Heart Study data. *CLUSTER* is among the best methods when the effects of causal variants are all in the same direction. As variants located in close proximity are more likely to have similar impact on disease risk, *CLUSTER* is recommended for association testing of clustered rare causal variants in case-control studies.

## Introduction

The development in next-generation sequencing technologies has allowed a comprehensive investigation of the role of rare variants (minor allele frequency (MAF) <1%) on complex diseases [1]. The low frequency of rare variants decreases the statistical power of detecting individual causal variants. Many statistical methods have been proposed to test for the collective association of multiple variants in a gene or region with diseases [2]–[13]. However, these methods do not incorporate the information of physical positions of the variants. Biological evidence suggests that multiple causal variants in a gene may cluster physically [14]. Variants within the same protein functional domain or gene regulatory element would locate in close proximity on the DNA sequence [15]–[18]. Furthermore, the spatial distribution of rare variants can be used to depict population structures [19]. These all constitute the importance of spatial approaches for rare variant association analyses.

Ionita-Laza et al. [14] has proposed a likelihood ratio scan statistic, and it successfully identifies clusters of rare deleterious variants with autism spectrum disorders. This method takes into account the underlying spatial distribution of variants, and we refer it to as “*IL-K*” because it is extended from the popular Kulldorff scan statistic [20]. It allows variable window sizes and calculates a likelihood ratio statistic for each window. The sliding window with the highest likelihood ratio statistic is the most likely region to harbor a cluster of rare deleterious variants. The statistical significance is assessed by permutation *P*-values [14].

Schaid et al. [16] has extended another popular spatial clustering method, Tango's statistic [21]–[23], to genomic sequence data. They incorporate the distance measures between variants into a kernel matrix, and therefore this method is referred to as “Kernel distance clustering” method (abbreviated as “*KERNEL*” hereafter). The statistic is 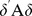, where 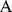 is the kernel matrix with spatial information, and 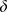 is the vector of case-control differences in variant frequencies. The statistical significance is also assessed by permutation *P*-values. Schaid et al. [16] have shown that *IL-K* outperforms *KERNEL* over a range of clustering scenarios, but *KERNEL* takes approximately half the computational time of *IL-K*.

We here propose a clustering method that is extended from the adaptive combination of *P*-values [24], [25]. This method truncates the variants with larger *P*-values which are more likely to be neutral variants. With extensive simulations, we have shown that our method outperforms *KERNEL* [16], the weighted-sum approach (referred to as “*WS*”) [4], and the variable threshold approach (referred to as “*VT*”) [6], in the majority of scenarios. It also outperforms *IL-K* [14] and the sequence kernel association test (*SKAT*) [8], [9] when all the causal variants are protective. We also apply this test to the Dallas Heart Study data [26], [27], to demonstrate its use in real data analyses.

## Materials and Methods

Suppose that there are *K* variant sites in a region of interest. We name the sites with larger variant frequencies in cases than in controls “deleterious-inclined variant sites”, and those with larger variant frequencies in controls than in cases “protective-inclined variant sites”. For a case-control study, the association of each variant with the disease status can be tested by the Fisher's exact test [13], [28] or by the logistic regression (if covariate adjustment is required). Let the per-site *P*-values of the *K* variants be 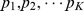, respectively. To test for the significance of the region, we combine the per-site *P*-values that are smaller than some truncation threshold. Suppose we consider *J* candidate truncation thresholds, 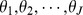.

Multiple causal variants may cluster spatially in a functional region [14]. The proposed method is extended from the adaptive combination of *P*-values [24], [25]. Furthermore, the spatial distribution of variants is taken into consideration. Under the *j*th truncation threshold (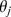), the significance signal accumulated by the deleterious-inclined variant sites is 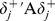, where 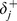 is a 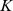-length vector with the *i*th element of 
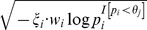
. The indicator variable 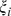 is 1 if the *i*th site is deleterious-inclined and 0 otherwise, 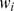 is the weight given to the *i*th site (detailed in the next paragraph), and 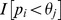 is 1 if the *P*-value of the *i*th site is smaller than the *j*th truncation threshold (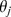) and 0 otherwise. Similarly, under the *j*th truncation threshold, the significance signal accumulated by the protective-inclined variant sites is 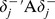, where 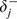 is a 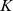-length vector with the *i*th element of 
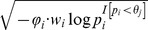
. The indicator variable 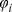 is 1 if the *i*th site is protective-inclined and 0 otherwise.

We follow Madsen and Browning [4] to determine the weights given to variant sites (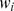
's). Let 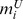 be the number of mutant alleles observed for variant *i* in the unaffected subjects, and let 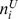 be the number of unaffected subjects genotyped for variant *i*. The frequency of variant *i* in the unaffected subjects is 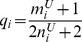. The weight given to the *i*th site is 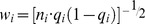, where 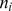 is the total number of subjects genotyped for variant *i*.

The 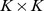 matrix 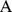 incorporates the spatial information of the variants. The (*i*, *j*)th element of 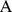 is 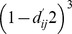, where 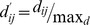, 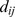 is the physical distance between the *i*th and the *j*th variants, and 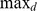 is a user-specified maximum distance of variants. Although the distance measure 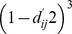 (named “tri-weight”) is used throughout this work, it can be replaced by other measures (see [16]).

Under the *j*th truncation threshold, a test statistic regardless of the directions of effects (deleterious or protective) is 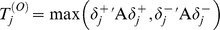. With *B* permutations by randomly shuffling the case/control status, we obtain the permuted statistics 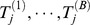. The *P*-value of the observed statistic 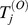 is estimated by 
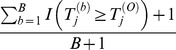
, and the *P*-value of the 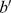
th permuted statistic 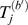 is estimated by 
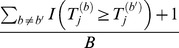
. Across the *J* candidate truncation thresholds, the minimum *P*-value of the observed sample is 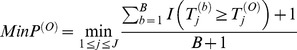, and the minimum *P*-value of the 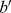
th permuted sample is 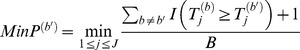. Because we have *B* permutations, we compare 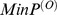 with 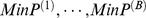, and the “adjusted *P*-value” is estimated by 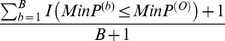. This method is referred to as “*CLUSTER*”, as it is proposed for detecting clusters of rare variants.

If we ignore the spatial information and let 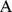 be an identity matrix (all the diagonal elements are 1 and all the off-diagonal elements are 0), the statistic 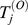 will be reduced to
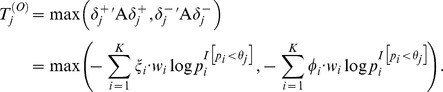



This is equivalent to the statistic of the “adaptive combination of *P*-values for rare variant association testing” (abbreviated as “*ADA*”) [24].

### Simulation Study

To simulate real human genomic structure, we used the Cosi program [29] that was based on a coalescent process [30]. We generated 100 data sets, each containing 10,000 chromosomes of 1 Mb regions. The chromosomes were generated according to the linkage disequilibrium patterns of the HapMap CEU (Utah residents with ancestry from northern and western Europe) samples [31]. For each data set, we randomly selected a ∼20 kb region. We considered two situations: (I) clustered causal variants: 20 rare causal variants were clustered within a ∼6 kb region; (II) non-clustered causal variants: 20 rare causal variants were approximately equally spaced across the whole ∼20 kb. The 20 causal variants were assumed to be (I) all protective; (II) 15 protective and 5 deleterious; (III) 10 protective and 10 deleterious; (IV) 5 protective and 15 deleterious; (V) all deleterious. The population attributable risk (PAR) of each causal variant was assumed to be 0%, 0.2%, 0.4%, 0.6%, 0.8%, and 1%, respectively.

Given PAR (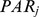) and MAF (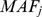) of the *j*th causal variant, its genotype relative risk (GRR) is:
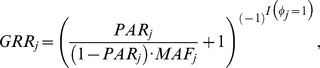

[4], [32]–[34]. The indicator function 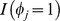 is 1 if the *j*th causal variant is protective, and is 0 otherwise. The genotypes of a subject were formed by two chromosomes randomly drawn from the pool of 10,000 chromosomes. For a subject with chromosomes 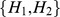, his/her disease status was generated by
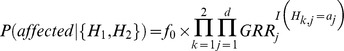

[32]–[34], where 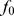 was the baseline penetrance (set at 1%), and 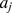 was the minor allele at the *j*th site. Chromosome pairs were randomly drawn from the chromosome pool with replacement until 500 cases and 500 controls were recruited.

### Tests under Comparison

We compared *CLUSTER* with *IL-K* [14], *KERNEL* [16], *SKAT* [8], [9], *WS* [4], and *VT* [6]. Single-nucleotide polymorphisms with MAF >5% in the combined sample of cases and controls were first removed from the analyses. The per-site *P*-values of individual variants were obtained by the mid *P*-values from the Fisher's exact test [28]. The user-specified maximum distance 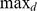 was fixed at 20 kb throughout this work. *IL-K* and *KERNEL* were implemented with the R package “vclust” [16]. The maximum window size considered by *IL-K* was set at 50% of the total region length, ∼10 kb, as suggested by Ionita-Laza et al. [14]. When performing “*KERNEL*”, tri-weight (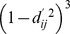) was used as the distance measure between any two variants, because this was the default setting in the R package “vclust” [16]. To have a fair comparison, *CLUSTER* was implemented with the same tri-weight distance measure. The candidate truncation thresholds considered in *CLUSTER* were 0.10, 0.11, 0.12, …, 0.20. These are suitable *P*-value truncation thresholds for rare variant association testing [24].

Two burden tests including *WS* and *VT* were implemented with the R script by Price et al. [6] (http://genetics.bwh.harvard.edu/rare_variants/). As a representative method of non-burden tests, *SKAT* was also included into comparisons. *SKAT* was implemented with the R package “SKAT” [35]. The weight given to the *j*th variant site (with MAF of 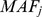) was set at 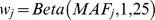, because this was the default weight function in the package “SKAT”. Note that the *SKAT* [9] compared here is the test that optimally combines the burden tests and the original *SKAT* proposed by Wu et al. [8].

The *P*-values of *CLUSTER*, *IL-K*, *KERNEL*, *WS*, and *VT* were obtained with 10,000 permutations when evaluating type-I error rates and 1,000 permutations when evaluating power, respectively. For *SKAT*, we used the default Davies method [36] in the package “SKAT” to compute *P*-values.

## Results

### Type-I Error Rates

The type-I error rates were measured when PAR was set at 0%. We performed 1,000 replications for each of the 100 simulated data sets. Therefore, there were totally 100,000 ( = 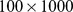) replications. Table 1 summarizes the type-I error rates given various nominal significance levels. The type-I error rates of all the six methods match the corresponding nominal significance levels.

**Table 1:** Type-I error rates.

nominal significance level	0.001	0.01	0.02	0.03	0.04	0.05
*SKAT*	0.0011	0.0102	0.0201	0.0303	0.0404	0.0503
*CLUSTER*	0.0008	0.0101	0.0204	0.0303	0.0401	0.0502
*KERNEL*	0.0011	0.0103	0.0187	0.0294	0.0404	0.0503
*IL-K*	0.0011	0.0100	0.0202	0.0298	0.0402	0.0501
*WS*	0.0008	0.0101	0.0200	0.0302	0.0404	0.0503
*VT*	0.0009	0.0100	0.0202	0.0304	0.0405	0.0502

### Power Comparisons

**Figure 1:**
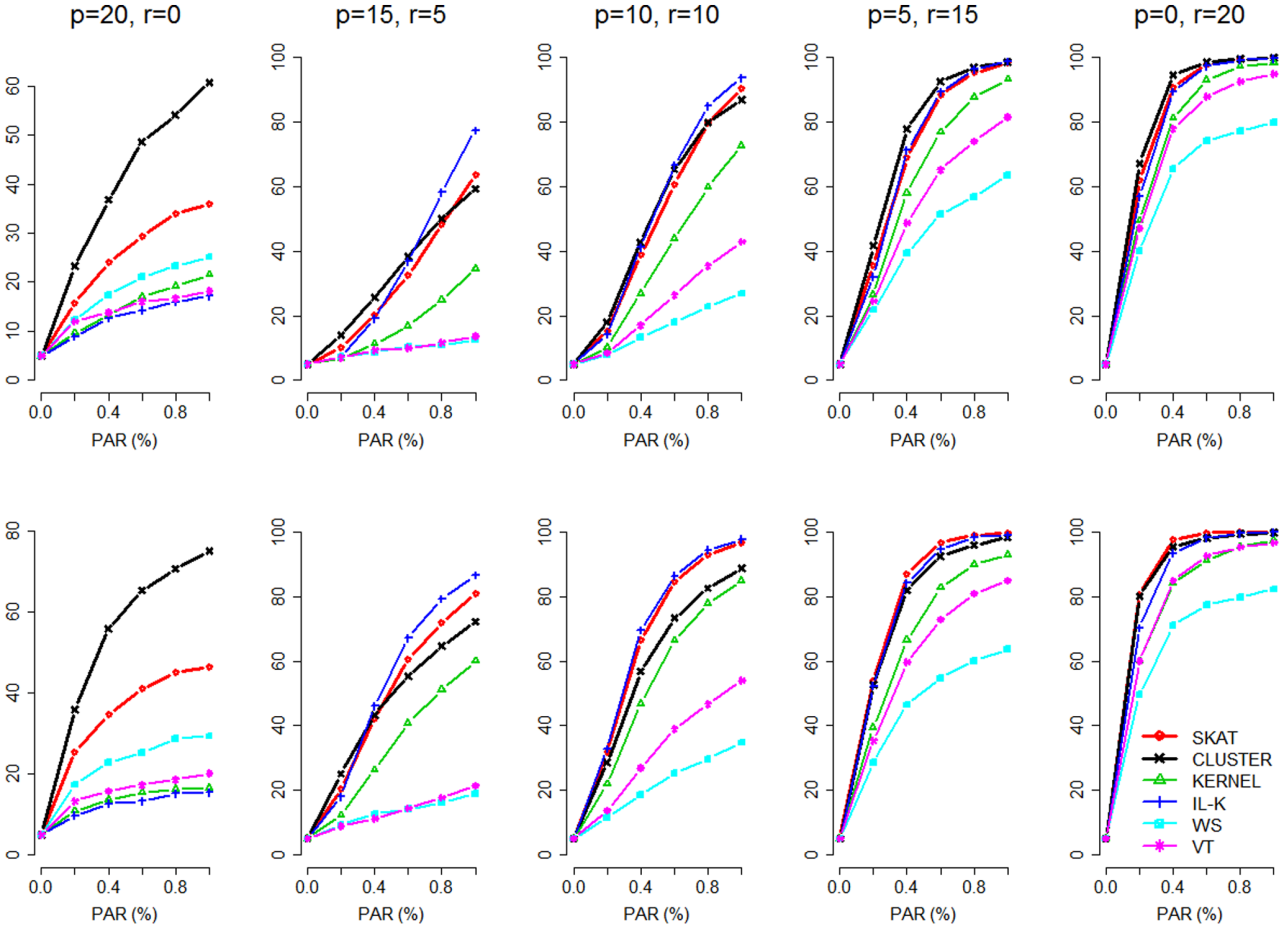
Simulation-Based Power Comparisons (20 rare causal variants). The figure shows the empirical power at 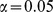. Top panel: clustered causal variants; bottom panel: non-clustered causal variants. The letters “p” and “r” denote the numbers of protective variants and deleterious (or, risky) variants, respectively.

To evaluate power, a total of 100 replications were performed under each scenario for each of the 100 simulated data sets. Figure 1 presents the power averaged over the 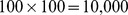 replications. When all the 20 causal variants were protective, *CLUSTER* was much more powerful than other methods. Under a mixture of deleterious and protective variants, *IL-K*, *SKAT*, and *CLUSTER* were powerful methods. However, *CLUSTER* had decreased power when the causal variants were non-clustered (see the bottom row). When all the 20 causal variants were deleterious, *IL-K*, *SKAT*, and *CLUSTER* were again the more powerful methods. Note that the effect size (measured by the magnitude of odds ratio) of a deleterious variant was larger than that of a protective variant with the same PAR and MAF (as shown by Lin et al. [24]). Therefore, all the methods performed better under 20 deleterious variants (the right column of Fig. 1) than under 20 protective variants (the left column of Fig. 1).

**Figure 2:**
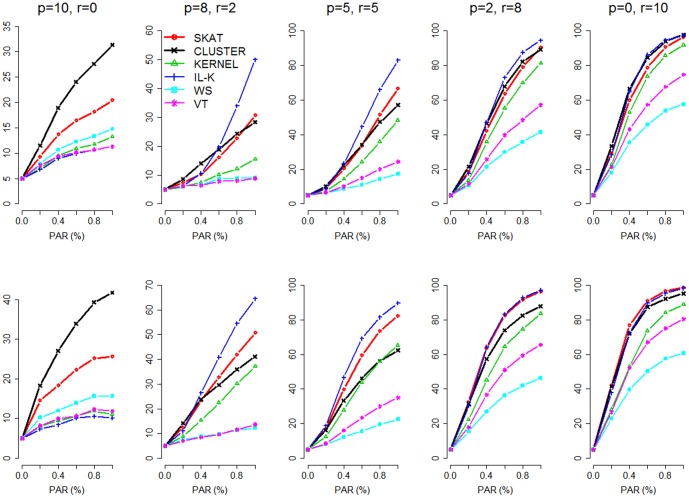
Simulation-Based Power Comparisons (10 rare causal variants). The figure shows the empirical power at 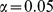. Top panel: clustered causal variants; bottom panel: non-clustered causal variants. The letters “p” and “r” denote the numbers of protective variants and deleterious (or, risky) variants, respectively.

We also evaluated the power performance of these tests when the number of causal variants was 10. Figure 2 shows the results of two situations considered: (I) clustered causal variants: 10 rare causal variants were clustered within a ∼3 kb region; (II) non-clustered causal variants: 10 rare causal variants were approximately equally spaced across the whole ∼20 kb. The 10 causal variants were assumed to be (I) all protective; (II) 8 protective and 2 deleterious; (III) 5 protective and 5 deleterious; (IV) 2 protective and 8 deleterious; (V) all deleterious. The result was similar to that shown by Fig. 1. *CLUSTER* was among the best methods when the effects of causal variants were all in the same direction, but it had decreased power under a mixture of deleterious and protective variants (see columns 2–4 of Figs. 1 & 2). This is because the test statistic 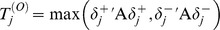 facilitates the detection of variants with effects in a consistent direction. We will further discuss this in the Discussion section.

In Figs. 1 and 2, the power from the top panel (clustered situation) is generally lower than that from the bottom panel (non-clustered situation). This is because, when the causal variants are clustered in a small region (∼6 kb or ∼3 kb, in the simulations), the variants far from this region will have almost no correlation (or, no linkage disequilibrium) with the causal variants. Therefore, they can hardly provide any association signal when testing for the whole region (∼20 kb here). When the causal variants are equally spaced across the whole region, the variants surrounding each causal variant can provide some signal because of their correlation with the causal ones. Although the correlation between rare variants is usually low [37], [38], it can still boost the power to some extent. This is a general trend for all the methods. What we can compare is the performance of the methods with spatial information (*CLUSTER*, *IL-K*, and *KERNEL*) relative to that of the methods without spatial information (*SKAT*, *WS*, and *VT*), in clustered situation or in non-clustered situation.

Not surprisingly, the clustered situation favors the methods considering spatial information (*CLUSTER*, *IL-K*, and *KERNEL*). They were relatively (relative to *SKAT*, *WS*, and *VT*) more powerful when the causal variants were clustered (top panels of Figs. 1 and 2). *CLUSTER* had good performance and was more powerful than *KERNEL*. *IL-K* also had good power performance, except when all the causal variants were protective (see the left columns of Figs. 1 and 2). With a mixture of protective and deleterious variants, *IL-K* was generally more powerful than *CLUSTER*, especially when the PAR was larger (see columns 2–4 of Figs. 1 and 2).

It was worth noting that *CLUSTER* outperformed *SKAT*, even when the causal variants were non-clustered (see the bottom-left plots of Figs. 1 and 2). This may be attributed to the “noise truncation” property of *CLUSTER*. The effect size (measured by the magnitude of odds ratio) of a protective variant was smaller than that of a deleterious variant with the same PAR and MAF (as shown by Lin et al. [24]). The effects of the protective variants were rather mild, and most methods were underpowered. *CLUSTER* takes the advantage of truncating neutral variants with larger *P*-values. *CLUSTER* is an extension of *ADA*, and this outcome is consistent with that observed in the *ADA* paper (see the left column of Fig. 2 of [24]).

### Application to Dallas Heart Study Data

These six tests were then applied to the Dallas Heart Study [26], [27], [39]. This study was to uncover the role of *ANGIOPOIETIN–LIKE 4* (*ANGPTL4*) in plasma triglyceride levels. The genotypes of 1,045 European Americans were analyzed. We first used a linear regression to adjust the log-transformed triglyceride levels for age, sex, and BMI. Subjects with residuals smaller than the 30^th^ percentile and larger than the 70^th^ percentile were treated as controls and cases, respectively. Then, the subjects with missing genotypes were excluded from the analysis. Finally, 179 cases and 213 controls were left.

The six tests were applied to this data set. The variants with MAF >5% were removed. To have an exhaustive search for the most likely region to harbor causal variants, the maximum window size considered by *IL-K* was set as the total region length (∼10 kb). As a result, only *CLUSTER* and *SKAT* had *P*-values smaller than 0.05 (see Table 2).

**Table 2:** Application to the Dallas Heart Study data.

	*SKAT*	*CLUSTER*	*KERNEL*	*IL-K*	*WS*	*VT*
*P*-value	0.0245	0.0125^a^	0.0899^a^	0.1398^a^	0.1841^a^	0.4858^a^

a *P*-values were obtained by 10,000 permutations.

The significant association of *ANGPTL4* with triglyceride was previously reported. Results in over 30,000 subjects from non-diabetic and population-based studies have confirmed that variants in *ANGPTL4* reduce triglyceride and exert protective effects against hyperlipidemia [26], [40], [41]. With the significance level of 0.05, only *CLUSTER* and *SKAT* confirmed this association. The other two spatial approaches, *IL-K* and *KERNEL*, were shown (by simulations) to have low power when all the causal variants were protective. No wonder they failed to detect the association here. This result is consistent with the finding from our simulation study.

## Discussion

Multiple rare variants may cluster in a functional region [14]–[18]. Variants within the same protein functional domain may locate in close proximity and have similar impact on disease risk [15], [17]. Consistent with the finding from Schaid et al. [16], *KERNEL* usually has lower power than *IL-K*. However, when all the causal variants are protective, *IL-K* has very low power (see the left columns of Figs. 1 & 2). This is because *IL-K* can only identify deleterious variants [14]. When all the causal variants are protective, *CLUSTER* and *SKAT* are more powerful than other methods. No wonder only these two methods could detect the protective effect of the variants in *ANGPTL4* against hyperlipidemia [26], [40], [41], in the Dallas Heart Study data analysis.

As mentioned in the Methods section, a test statistic regardless of the directions of effects (deleterious or protective) is 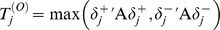 under the *j*th truncation threshold. Another reasonable statistic is 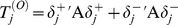. This is more powerful than *CLUSTER* when ∼50% of the causal variants are deleterious, but is less powerful when the effects of variants are all in the same direction. Because clustered variants are more likely to have effects in the same direction, we still suggest using 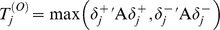, instead of 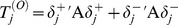. Note that even the statistic, 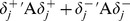, is started from aggregating the information of “deleterious-inclined variants” and “protective-inclined variants”, separately. Under the assumption that deleterious variants and protective variants may have their own clusters, we do not mix all the variants together in the very beginning (i.e., 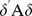, this will incorporate the distance between “deleterious-inclined variants” and “protective-inclined variants” into the statistic).

All the methods evaluated here require permutations to obtain accurate *P*-values, except *SKAT* that uses the Davies method [36] to compute *P*-values. For simulated data sets each containing 500 cases and 500 controls in ∼20 kb regions (including ∼330 nonsynonymous variant sites), the computation time lengths were ordered as *CLUSTER* (∼151.7 sec) > *SKAT* (∼30.2 sec) > *IL-K* (∼20.4 sec) > *KERNEL* (∼6.7 sec) > *VT* or *WS* (∼3.4 sec), where 1000 permutations were used for all the methods except *SKAT*. This was timed by a Linux workstation with an Intel Xeon E5-2690 2.9 GHz processor and 6 GB memory. *CLUSTER* takes a longer time to compute because it incorporates the spatial kernel matrix into the search of the optimal *P*-value truncation threshold.

Schaid *et al.* [16] showed that *IL-K* and *KERNEL* could have higher power than *SKAT*, when the variants were correlated. Without correlation, *SKAT* tended to have the highest power among the tests they compared [16]. In fact, the correlation between rare variants is usually low [37], [38]. Our simulated data sets were generated from the coalescent process [30] and they reflected realistic DNA sequences. Therefore, in our simulations, the correlation between rare variants is low and *SKAT* is better than *KERNEL* (and sometimes better than *IL-K*).

*KERNEL* and *CLUSTER* have similar forms in test statistics (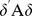), and they are both implemented with the tri-weight distance measure in our simulations. However, the results showed that *CLUSTER* outperformed *KERNEL*. This is because *CLUSTER* combines the association signals (*P*-values) of variants that are more likely to be causal, i.e., truncates variants with larger *P*-values. *CLUSTER* is among the best methods when the effects of causal variants are in one direction. As variants located in close proximity are more likely to have similar impact on disease risk [15], [17], *CLUSTER* is recommended for association testing of clustered rare causal variants in case-control studies.
